# Genomic and Phenotypic Characterization of *Streptomyces marxii* sp. nov., Producer of Kinanthraquinone B

**DOI:** 10.3390/microorganisms14061206

**Published:** 2026-05-27

**Authors:** Mikhail Yu. Dobryakov, Julia A. Buyuklyan, Mikhail V. Biryukov

**Affiliations:** 1 Research Center for Translational Medicine, Sirius University of Science and Technology, Sochi 354340, Russia; dobryakov.my@gmail.com (M.Y.D.); buyuklyan20@gmail.com (J.A.B.); 2 Department of Biology, Lomonosov Moscow State University, Moscow 119991, Russia

**Keywords:** *Streptomyces*, kinanthraquinone B, pDualrep2, BGCs, polyphasic taxonomy, new species, *Actinomycetota*, topoisomerase catalytic inhibitors

## Abstract

Describing novel microbial species opens access to uncharted biosynthetic gene clusters and their associated secondary metabolites, offering fresh opportunities in the search for new antibiotics urgently needed to combat multidrug resistance. In this study, we describe a new species of *Streptomyces*, *S. marxii* sp. nov. (type strain VKM Ac-3100), an actinobacterium isolated from soil in the Yaroslavl Region of Russia. Using a polyphasic taxonomic approach that included whole-genome sequencing (WGS), we found that the strain’s average nucleotide identity (ANI) and digital DNA–DNA hybridisation (dDDH) values relative to its closest relative, *S. maoxianensis*, were 92.53% and 47.9%, respectively. Both values fell significantly below the species delimitation thresholds. Functional screening using the pDualrep2 dual fluorescent reporter system identified a unique SOS-silent antimicrobial profile characterised by growth inhibition without induction of the SOS response or translation stress. High-resolution mass spectrometry (HRMS) and genomic mining revealed that this activity is linked to the production of kinanthraquinone B ([M+H]^+^ *m*/*z* 275.0550), a rare polycyclic aromatic polyketide. Genomic analysis identified a specialised type II polyketide synthase (T2PKS) biosynthetic gene cluster (BGC) with evidence of acquisition via horizontal gene transfer (HGT). Our findings characterise *S. marxii* as a promising natural producer of rare catalytic inhibitors of DNA topoisomerases II and IV, offering a scaffold for the development of antibiotics with potentially lower genotoxicity.

## 1. Introduction

The current global health crisis, caused by the rapid spread of multidrug-resistant (MDR) pathogens, has made it critically important for global medicine to find new antimicrobial agents [1]. According to the most recent projections presented as part of the Global Burden of Antimicrobial Resistance project, by 2050 the number of deaths per year directly attributable to antibiotic resistance could reach 1.91 million [2]. At the same time, the total number of deaths caused by bacteria resistant to antibiotics will exceed 39 million people. The economic damage from the decline in the effectiveness of antimicrobial therapy is estimated at hundreds of billions of US dollars a year. This shows that there is an urgent need to intensify programmes for the search for new natural compounds [3]. Representatives of the phylum *Actinomycetota*, and in particular the genus *Streptomyces* (family Streptomycetaceae, order Kitasatosporales), remain the most reliable source for discovering new scaffolds for active molecules [4]. Since streptomycin was discovered in 1943, streptomycetes have been shown to be exceptional natural producers, responsible historically for about 75% of all known natural antibiotics [5].

The genus *Streptomyces* comprises Gram-positive filamentous bacteria with a complex developmental cycle involving the formation of vegetative and aerial mycelium followed by differentiation into spore chains [6]. Members of this group carry a linear chromosome between 6 and 12 Mb in size. The genomic DNA is GC-rich, with values typically ranging from 69% to 78% [7]. The genetic variability of streptomycetes, driven by horizontal gene transfer and gene duplication, allows them to encode between 25 and 70 secondary-metabolite biosynthetic gene clusters (BGCs) per genome [8], but up to 90% of these clusters remain cryptic under standard laboratory conditions, which requires the development of new methods for their activation [9,10]. Current estimates indicate that the characterised chemical space of the genus represents only a small fraction of its total biosynthetic capacity. Projections suggest that over 150,000 antimicrobial molecules remain undiscovered in unexplored natural strains [11].

Kinanthraquinones are a class of anthraquinone carboxamides produced primarily by *Streptomyces* species. They are typically synthesised by iterative type II polyketide synthase (T2PKS) systems that assemble complex, multi-ring structures. Similar structures have been observed in kinanthraquinones B and D. Kinanthraquinones exhibit antibacterial, cytotoxic and antimalarial activity [12]. A distinctive pharmacological feature of some kinanthraquinones is their mechanism of action as catalytic inhibitors of bacterial DNA topoisomerases II and IV [13]. Unlike fluoroquinolones, which cause lethal DNA damage and trigger the SOS response, catalytic inhibitors arrest growth without activating standard reporter signals, such as TurboRFP, in systems like pDualrep2, producing an SOS-silent phenotype. The bulky structure of many kinanthraquinones limits their diffusion across the intact outer membrane of Gram-negative bacteria, necessitating the use of permeability mutants such as *E. coli lptD* for detection.

Traditionally, soil has been the main source of new streptomycete species [14]. In this respect, Russia, with its enormous diversity of soil types, represents a rich reservoir of microbial biodiversity [15]. Modern methods of whole-genome sequencing and bioinformatic analysis (ANI, dDDH) have refined the taxonomic landscape, replacing earlier chemotaxonomic approaches with genome-based metrics [16]. Modern systems like the Type Strain Genome Server (TYGS) can be used to precisely identify new species based on their genomic differences [17,18]. Also, the introduction of highly sensitive reporter systems, such as pDualrep2, allows the mechanism of action of metabolites to be determined at an early stage [19]. The combination of genomic mining and functional screening methods opens up new opportunities for identifying producers of rare antibiotics and accelerating the dereplication of known compounds [20].

In this paper, we present the results of a polyphasic study of the *S. marxii* strain isolated from soils in the Yaroslavl Region, Russia. The strain was identified as a new species, *S. marxii* sp. nov., based on whole-genome analysis combined with phenotypic, chemotaxonomic and functional characterisation.

## 2. Materials and Methods

### 2.1. Collection, Isolation, and Preservation

*S. marxii* was isolated from a soil sample collected in the Yaroslavl Region, Russia (57.188722° N, 39.418857° E). Pure cultures were obtained by repeated subculturing and subsequently maintained on oat agar (ISP 3) [21]. Long-term storage was carried out at −80 °C in 20% (*v*/*v*) glycerol.

### 2.2. Phenotypic and Biochemical Characteristics

#### 2.2.1. Morphological and Cultural Properties

The morphological and cultural properties of the isolate were studied on standard media of the International *Streptomyces* Project (ISP 2, ISP 3, ISP 4, ISP 5, ISP 6 and ISP 7) [22]. Inoculation was performed with a bacterial suspension obtained after 7 days of growth in ISP 3 liquid medium at 28 °C with shaking New Brunswick Innova^®^ 44/44R (Eppendorf, Framingham, MA, USA) 28 °C, 160 rpm, 7 days). After 14 days, the colour of the aerial mycelium on the surface was assessed using the RAL colour scale.

The morphology of cells, spore-bearing structures and spore surfaces was examined using scanning electron microscopy (SEM) on a JSM-6380LA instrument (JEOL Ltd., Akishima, Tokyo, Japan). Samples for SEM were prepared by fixing 2.5% agar blocks with glutaraldehyde, followed by dehydration in a graded ethanol series and critical-point drying.

#### 2.2.2. Carbon Source Utilisation

The ability to utilise various carbon sources was determined using ISP medium 9, based on the Pridham–Gottlieb basal medium, as the basal mineral medium [23]. The carbohydrates tested included glucose, mannitol, sucrose, arabinose, galactose, inositol, cellobiose, xylose, lactose, maltose, mannose, rhamnose, raffinose, fructose, and salicin. Growth was assessed visually by comparison with a negative control (without carbon) and a positive control (with glucose).

To ensure accurate results and to prevent carry-over of nutrients from the primary medium, bacterial biomass was harvested from ISP 3 liquid culture by centrifugation (4000× *g* for 10 min), washed three times with sterile saline (0.85% NaCl) and resuspended to a final OD_600_ of 0.1. The tested carbohydrates were prepared as 10% (*w*/*v*) stock solutions, sterilised by filtration through 0.22 µm pore-size membranes and added to the autoclaved ISP 9 base to a final concentration of 1% (*w*/*v*). The plates were incubated at 28 °C for 14 days. Growth was assessed visually by comparison with two controls: a negative control (ISP 9 without any carbon source) and a positive control (ISP 9 supplemented with D-glucose). Results were recorded as follows: abundant growth (equal to or greater than the positive control), slight growth (significantly better than the negative control but less than the positive control), or no growth (equal to the negative control). The carbohydrates tested included D-glucose, D-mannitol, sucrose, L-arabinose, D-galactose, myo-inositol, cellobiose, D-xylose, lactose, maltose, D-mannose, L-rhamnose, D-raffinose, D-fructose and salicin [24].

#### 2.2.3. Physiological and Biochemical Assays

Antibiotic sensitivity was determined using the disc-diffusion method on Mueller–Hinton medium. Growth was assessed at different temperatures (8, 15, 20, 28, 37, 45 and 50 °C) and at different NaCl concentrations (1%, 5% and 8% *w*/*v*), and the pH range for growth was determined in Organic Broth 79 within the tested range of pH 4.5 to 9.5 after two weeks of incubation, as previously described [25]. Hydrolytic enzyme activities were assessed on solid media: amylase activity on starch agar (1% soluble starch) revealed by iodine staining, protease activity on skim-milk agar and nuclease activity on DNA agar (Difco) with toluidine-blue flooding. All susceptibility tests were performed in triplicate (*n* = 3) using independent bacterial cultures to ensure statistical reliability. The results are presented as the mean diameter of the inhibition zones (mm) ± standard deviation.

### 2.3. Genome Sequencing, Phylogenomic and Bioinformatic Analysis

Genomic DNA was extracted from a pure culture using the ZymoBIOMICS DNA Microprep Kit (Zymo Research, Irvine, CA, USA). Paired-end library preparation was performed using the NEBNext Ultra II DNA Library Prep Kit (New England Biolabs, Ipswich, MA, USA)). Sequencing was performed on the Illumina MiSeq platform (MiSeq Reagent Kit v3, 600 cycles). Primary processing of reads (quality filtering Q > 20 and adapter removal) was performed in Fastp v1.3.3 [26]. De novo genome assembly was performed in SPAdes v3.15.5 in careful mode [27]. Assembly quality was assessed using BUSCO v5.5.0 (actinobacteria_class_odb10 database) [28] and CheckM v1.2.4 [29]. For phylogenetic analysis, the 16S rRNA gene sequence was extracted from the genome using RNAmmer [30] and compared with type strains from the TYGS database [17]. Alignment was performed using ClustalW version 2.1 [31]. The phylogenetic tree was constructed using FastME 2.1.6.1 [32] based on Genome BLAST Distance Phylogeny (GBDP) distances generated by the TYGS/GGDC pipeline [17,33]. Branch stability was assessed using 100 pseudo-bootstrap replicates. The search for biosynthetic gene clusters (BGCs) was performed in antiSMASH 8.0.4 [34]. The ClusterCompare option was used to search the MIBiG 4.0 database for similarities [35]. BGC alignment was performed using the clinker tool version 0.0.32 [36].

### 2.4. Testing Antibacterial Activity on Reporter Strains

To determine the mechanism of action of *S. marxii* antibacterial metabolites, the pDualrep2 dual-reporter system was used [19]. This system is based on the use of the *E. coli* JW5503 strain carrying a Δ*tolC* deletion and an *E. coli* strain with a mutation in *lptD*, both characterised by a disrupted outer-membrane barrier function. Both strains carry genes for fluorescent proteins whose expression is induced by specific stress responses of the cell. Induction of Katushka2S expression serves as an indicator of translation inhibition, while TurboRFP expression indicates activation of the SOS response due to DNA damage or replication defects.

To confirm that the observed antimicrobial activity was due to specific metabolites rather than the synergistic effect of a crude mixture, testing was performed using both the culture supernatant and individual HPLC-purified fractions. All assays were conducted in triplicate (*n* = 3) to confirm the stability of the inhibition zones and the consistency of the reporter response.

Testing was performed using the agar-diffusion method. Samples of culture fluid or extracts in a volume of 100 µL were applied to wells on the surface of the reporter strain lawn. Plates were incubated at 37 °C for 12–18 h, after which the fluorescent signal was recorded in the Cy3 (TurboRFP) and Cy5 (Katushka2S) channels. Norfloxacin and erythromycin were used as positive controls. Antifungal activity was additionally evaluated against *Candida albicans* ATCC 10231 by the agar well-diffusion method on Sabouraud dextrose agar, using amphotericin B (10 µg/disc) as a positive control and incubating at 30 °C for 48 h.

### 2.5. Purification and Identification

The purification process followed a bioactivity-guided approach, whereby only the fractions exhibiting the distinctive SOS-silent inhibition phenotype (growth inhibition without SOS induction) were selected for subsequent HRMS identification. This approach enabled us to isolate the specific compound responsible for the diagnostic biological effect.

Primary purification of antimicrobial compounds from the culture fluid of *S. marxii* was performed by solid-phase extraction. Hydrophobic polyvinylbenzene LPS-500-H (pore size 50–1000 Å) was used as the sorbent. Elution of the target components was performed using a stepwise gradient of aqueous acetonitrile (0, 10, 20, 30, 40, 50, 75 and 100%). The activity of the obtained fractions was analysed using the pDualrep2 reporter system. The fractions eluted with 20% and 30% acetonitrile showed the highest inhibitory activity and were selected for detailed analysis.

HPLC analysis and fractionation were performed on a Vanquish Flex UHPLC system (Thermo Fisher Scientific, Waltham, MA, USA) with a diode-array detector equipped with a Luna C18(2) column (5 µm, 100 Å, 250 × 4.6 mm; Phenomenex, Torrance, CA, USA). For high-resolution mass spectrometry, a maXis II 4G ETD mass spectrometer (Bruker Daltonics, Bremen, Germany) coupled with an UltiMate 3000 chromatograph (Thermo Fisher Scientific, Waltham, MA, USA) was used. Separation was performed on an Acclaim RSLC 120 C18 analytical column (2.2 µm, 2.1 × 100 mm). Mass spectra were recorded in ESI+ mode (positive ionisation) with full scanning in the range *m*/*z* 100–1500. Tandem mass spectrometry (MS/MS) was performed for the three most intense ions using CID fragmentation (collision energy 10–40 eV) and nitrogen as the collision gas. Data were processed using Bruker Compass DataAnalysis 5.2.

### 2.6. Statistical Analysis

To ensure the validity of the presented data, a multifaceted statistical approach was employed. For phenotypic assays, including antibiotic susceptibility and growth-range studies, experiments were performed in triplicate (*n* = 3), with results expressed as the mean ± standard deviation. Genomic and taxonomic reliability was established using standardised bioinformatic metrics. The quality of the *S. marxii* genome assembly was statistically validated using BUSCO v5.5.0 to assess completeness and CheckM v1.2.4 to determine contamination and strain heterogeneity. Phylogenetic tree stability was confirmed through 100 pseudo-bootstrap replicates. Genomic distances, including dDDH and ANI, were calculated using the TYGS platform, which utilises robust algorithmic frameworks to provide high-confidence species delimitation. These metrics collectively form the statistical basis for validating the description of the novel species.

## 3. Results and Discussion

### 3.1. Morphological and Cultural Characteristics

**Figure 1:**
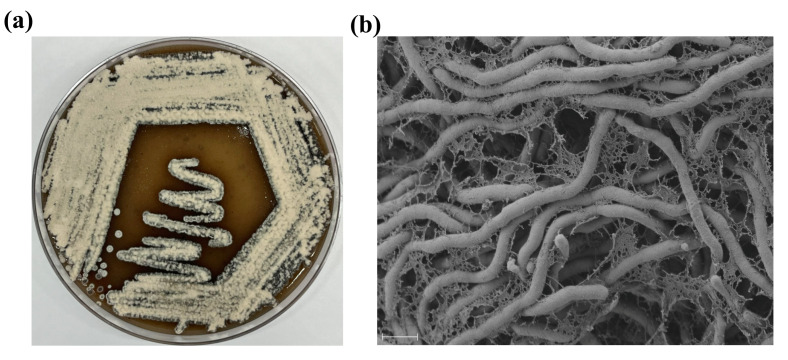
(**a**) Cultural and morphological properties of *S. marxii* on ISP3 media after 14 days at 28 °C; (**b**) Scanning electron micrograph of *S. marxii* grown on ISP 3 agar for 14 days at 28 °C. Imaging condi-tions: magnification 10,000× (EHT = 1.00 kV; working distance = 3.1 mm; SE2 detector; probe current = 100 pA; system vacuum = 1.50 × 10^−6^ mbar). Scale bar, 1 μm.

*S. marxii* showed classic characteristics of the genus *Streptomyces*, forming well-developed substrate and aerial mycelium. On ISP 3 medium (oat agar), the aerial mycelium had a greyish-white colour (RAL 9002), while the substrate mycelium was olive-brown (RAL 8008) (Figure 1a). On most of the tested media, pigmentation ranged from beige to light brown. Detailed data on pigmentation on standard media are given in Table 1. Microscopic examination of strain *S. marxii* revealed long, branched mycelial filaments with a diameter of approximately 0.5–1.0 µm. Spore chains formed on aerial mycelium and contained 10–50 spores (Figure 1b). Cell motility was not observed, and no streaks were formed. The strain is Gram-positive and catalase-positive. These morphological characteristics are broadly consistent with those reported for *S. maoxianensis* [37], which similarly forms grey aerial mycelium and olive-brown substrate mycelium on ISP media. However, *S. marxii* is distinguished by more pronounced pigment development on ISP 5 and ISP 7 media (pearl gold and ochre brown, respectively), which was not reported for *S. maoxianensis*. Together with the genomic data, these morphological differences further support the classification of the isolate as a distinct species.

### 3.2. Physiological and Biochemical Properties

Strain *S. marxii* is a mesophilic organism with growth occurring between 15 °C and 37 °C and an optimum around 28 °C; no growth was observed at 8 °C or at 45 °C and above within the tested range. The strain behaved as a neutrophile, demonstrating best growth at pH 7.0 with a growth range of 5.5–8.5 within the tested range of pH 4.5–9.5. An important characteristic is salt tolerance: *S. marxii* was capable of growing in the presence of NaCl at concentrations up to 5% (*w*/*v*), with optimal growth observed at 1% among the three concentrations tested (1%, 5% and 8%).

The ability of *S. marxii* to utilise carbon sources was compared with data for its closest phylogenetic relative, *S. maoxianensis*. The results are presented in Table 2.

Strain *S. marxii* demonstrated a broader range of metabolic capabilities compared to *S. maoxianensis*, successfully assimilating mannitol, sucrose and arabinose. Nuclease, protease and amylase activities were detected on the corresponding solid media (see Section 2.2.3), indicating the presence of additional hydrolytic enzyme systems, which may be related to adaptation to the organic-rich loam soils of the Yaroslavl Region.

The antibiotic susceptibility profile of *S. marxii* was determined using the disc-diffusion method on Mueller–Hinton medium. The isolate showed sensitivity to several clinically significant antibiotics, including fluoroquinolones (levofloxacin: 15.0 ± 0.7 mm; norfloxacin: 13.0 ± 0.5 mm; ciprofloxacin: 10.0 ± 0.4 mm), glycopeptides (vancomycin: 21.5 ± 1.5 mm) and aminoglycosides (amikacin: 20.0 ± 0.9 mm; gentamicin: 14.0 ± 0.6 mm; tobramycin: 14.0 ± 0.5 mm). High sensitivity was observed for the combination of trimethoprim/sulfamethoxazole (27.0 ± 1.3 mm) and for tigecycline (14.0 ± 0.7 mm). At the same time, the strain showed resistance to most of the tested β-lactams (oxacillin, ampicillin, cefepime, ceftazidime, cefoxitin), lincosamides (clindamycin), azithromycin, and to erythromycin at 5 µg disc content. The observed sensitivity to fluoroquinolones is consistent with the identification of DNA topoisomerases II and IV as the primary targets of kinanthraquinone B in this strain: fluoroquinolones and catalytic topoisomerase inhibitors share the same molecular target, though they differ fundamentally in their mechanism of action, the former stabilising cleavage complexes while the latter blocking catalytic turnover. The intrinsic resistance to β-lactams and macrolides (erythromycin, azithromycin) is a well-documented characteristic of the genus *Streptomyces*, attributable to the absence of the penicillin-binding protein targets in the thick peptidoglycan wall and to chromosomally encoded efflux systems, and is therefore not diagnostically informative at the species level.

### 3.3. Phylogenetic Analysis

**Figure 2:**
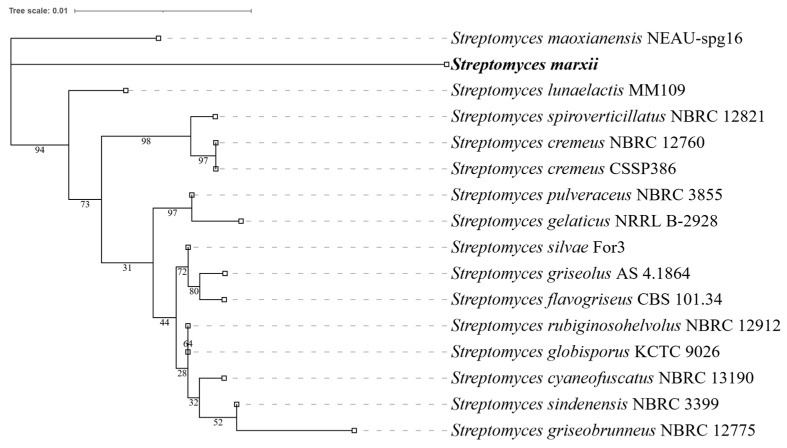
Phylogenetic tree of *S. marxii* and type strains of the related species. The strain described in this study is shown in bold.

TYGS analysis confirmed that *S. marxii* belongs to the genus *Streptomyces*. Phylogenetic analysis of the 16S rRNA gene (1519 bp, GenBank accession PV849038) showed that *S. marxii* forms a separate branch, closest to the cluster of *S. maoxianensis* and *S. globisporus* (Figure 2).

In the phylogenetic tree constructed based on the 16S rRNA gene sequence, *S. marxii* is located in the same cluster as the type strains *S. maoxianensis*, *S. lunaelactis* and *S. achmelvichensis*. Quantitative indices of whole-genome comparison indicate a substantial genetic distance between the studied strain and its closest relatives. The results of dDDH analysis are presented in Table 3.

The generally accepted threshold for distinguishing between bacterial species is 70% dDDH. The value of 47.9% for the pair *S. marxii*/*S. maoxianensis* indicates that the strain under study represents a new species. Despite the 16S rRNA gene sequences being very similar (>98%), the genomic indices show substantial differences [37]. The ANI value (92.53%) is also below the threshold of 95–96% for species differentiation. This highlights the limitations of using only 16S rRNA for the taxonomy of streptomycetes, whose genomes are subject to intense recombination and horizontal gene transfer [38]. This discordance between high 16S rRNA similarity and substantial genome-level divergence is not unusual in the genus *Streptomyces*: ribosomal genes are among the most conserved in the bacterial genome, and their sequence identity can remain above 98–99% between species that are clearly separated by all other genomic and phenotypic criteria [38]. The dDDH value of 47.9% places *S. marxii* and *S. maoxianensis* clearly within separate species, and the genetic distance to all other type strains in Table 3 (dDDH 23–34%) confirms that *S. maoxianensis* is the sole closest relative, and that *S. marxii* cannot be assigned to any previously described species. This combination of evidence provides an unambiguous basis for the description of a novel species.

The genome size of *S. marxii* is approximately 8.9 Mb with a G+C content of 69.6 mol%, which is consistent with the parameters of related species. The genome is predicted to contain 8211 protein-coding sequences. The high quality of the assembly was confirmed by BUSCO (C: 99.7% [S: 98.3%, D: 1.4%], F: 0.3%, M: 0.0%) and CheckM (completeness 99.29%, contamination 1.80%). The elevated CheckM contamination value most likely reflects paralogous gene families characteristic of large *Streptomyces* genomes rather than true contamination, which is consistent with the 1.4% duplication detected by BUSCO and with the absence of significant missing or fragmented markers. The *S. marxii* genome assembly has been deposited in GenBank under the accession number SAMN57460202.

### 3.4. In Silico Chemotaxonomy and Genomic Mining

Analysis of the annotated *S. marxii* genome revealed a complete set of genes characteristic of the *Streptomyces* chemotype.

Fatty acids. A conserved operon for type II fatty acid biosynthesis (FAS II) was identified, comprising *fabD* (malonyl-CoA:ACP transacylase), *fabH* (β-ketoacyl-ACP synthase III), *acpP* (acyl carrier protein) and *fabF*. Multiple copies of *fabG* (3-oxoacyl-ACP reductase) were also identified, consistent with the ability to synthesise iso- and anteiso-type saturated fatty acids.

Cell wall components. The complete biosynthetic pathway of LL-diaminopimelic acid (LL-DAP) from aspartate was identified, including the key genes *dapA*, *dapB*, *dapC*, *dapD*, *dapE* and *dapF*. The presence of *murE* (UDP-N-acetylmuramoyl-L-alanyl-D-glutamate:2,6-diaminopimelate ligase) confirms incorporation of LL-DAP into the peptidoglycan.

Polar lipids. The genome contains *cdsA* (phosphatidate cytidylyltransferase, CDP-diacylglycerol synthase), *pgsA* (phosphatidylglycerophosphate synthase) and *clsA* (cardiolipin synthase), consistent with the presence of diphosphatidylglycerol (DPG), phosphatidylethanolamine (PE) and phosphatidylinositol (PI) in the membrane, typical for *Streptomyces*.

Respiratory quinones. Genes involved in the biosynthesis of menaquinones, including *menG* (methyltransferase) and *menJ* (menaquinone reductase), were identified, consistent with the production of hydrogenated menaquinones of the MK-9 series.

Although modern in silico analyses can reliably predict the presence of chemotaxonomic markers such as LL-DAP and branched-chain fatty acids, these predictions should be considered high-probability indications and be complemented by classical analytical chemistry where possible. The predicted chemotaxonomic profile of *S. marxii*—LL-DAP cell wall, MK-9(H_6_)/MK-9(H_8_) menaquinones and iso-/anteiso-branched fatty acids—is consistent with the chemotype I characteristic of the genus *Streptomyces* and matches the reported chemotaxonomic data for *S. maoxianensis* [37], confirming that *S. marxii* is correctly placed within the genus on chemotaxonomic grounds, and that the species-level distinction rests on genomic and functional criteria rather than chemotaxonomic divergence.

### 3.5. Antimicrobial Activity

**Figure 3:**
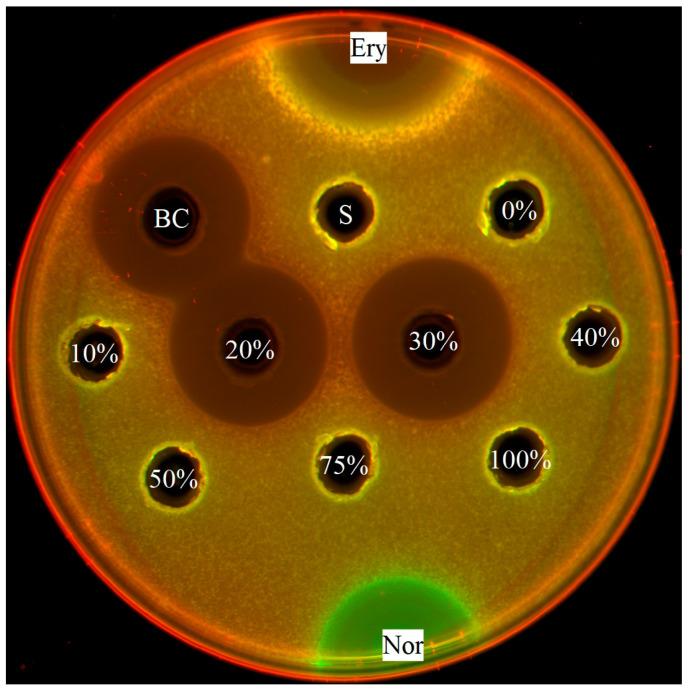
Inhibition of growth of *E. coli lptD*. Control antibiotics: erythromycin (Eri) and norfloxacin (Nor). The fluorescence of the *E. coli* lawn was scanned at 553/574 nm (TurboRFP) and 588/633 nm (Katushka2S). Induction of Katushka2S expression is triggered by translation inhibitors, while TurboRFP is regulated by the SOS response to DNA damage. BC—broth culture of *S. marxii*; S—supernatant; 0, 10, 20, 30, 40, 50, 75 and 100%—elution with aqueous acetonitrile solutions of the indicated volume concentrations.

Extracts of *S. marxii* exhibited a highly specific activity phenotype. The metabolites effectively inhibited the growth of *E. coli lptD* but remained completely inactive against *E. coli* Δ*tolC* and wild-type strains. No induction of either Katushka2S or TurboRFP was observed within the inhibition zone of the *E. coli lptD* strain (Figure 3). This indicates that the active component is neither a classical translation inhibitor nor a direct DNA-damaging agent. The lack of activity against *E. coli* Δ*tolC* despite the effect on *E. coli lptD* suggests that the active molecule is bulky or highly hydrophobic, preventing its entry through an intact Gram-negative outer membrane. *S. marxii* showed no antagonism against *Candida albicans*, suggesting selectivity towards prokaryotic targets, although testing against a broader panel of fungi would be required to confirm this.

### 3.6. Identification of the Antibacterial Compound and Biosynthetic Gene Cluster

**Figure 4:**
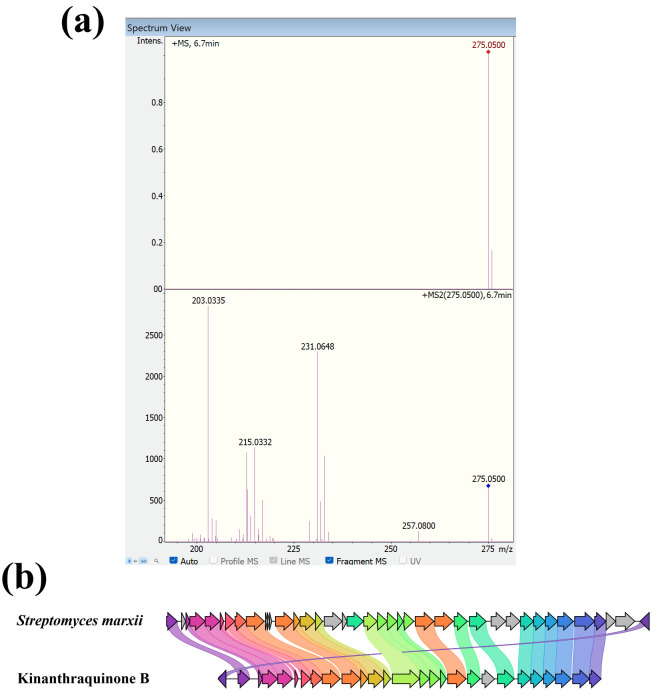
(**a**) High-resolution MS and MS^2^ fragmentation spectrum of the [M+H]^+^ ion (*m*/*z* 275.0550) of the iso-lated compound; the y-axis shows signal intensity in arbitrary units (×10^6^), and MS^2^ spectra were acquired at a collision energy of 24.4–36.6 eV. (**b**) Comparison of the reference kinanthraquinone B cluster (BGC0002383) with the corresponding region of the S. marxii genome, generated using clinker [36]; coloured links connect homologous genes, with colour intensity proportional to the pairwise ami-no-acid identity between them.

Genomic profiling linked to mass spectrometry identified the active principle as a member of the kinanthraquinone family. Genome analysis of *S. marxii* revealed a T2PKS cluster with approximately 61% overall similarity (antiSMASH ClusterCompare) to the reference kinanthraquinone B cluster (BGC0002383 [13]) from *Streptomyces* sp. SN-593. Comparative genomic analysis using the clinker tool confirmed notable synteny between the core PKS and tailoring enzyme genes of *S. marxii* and those of BGC0002383 (Figure 4a).

HRMS analysis of the active fractions at *t*_R_ 6.70 min detected a peak at *m*/*z* 275.0550 corresponding to [M+H]^+^ of kinanthraquinone B (C_14_H_10_O_6_, calculated 275.0550). MS^2^ fragmentation showed characteristic neutral losses consistent with the anthraquinone core: *m*/*z* 257.05 ([M+H−H_2_O]^+^), 231.06 ([M+H−H_2_O−CO]^+^) and a core fragment at *m*/*z* 203.03 (Figure 4b). While NMR spectroscopy remains the definitive method for absolute structural elucidation of novel natural products, the identification of kinanthraquinone B in this study relies on a robust dereplication framework. The exact molecular formula, determined by HRMS, combined with the characteristic MS^2^ fragmentation pattern, the notable synteny between the *S. marxii* BGC and the reference kinanthraquinone B cluster, and the diagnostic SOS-silent topoisomerase inhibition phenotype, collectively provide a high level of confidence for the identification of this metabolite within the context of a species description. Further NMR-based structural confirmation is planned as part of a future study focused on the complete chemical profiling of the *S. marxii* metabolome.

The absence of a homologous biosynthetic pathway in the closest phylogenetic neighbour *S. maoxianensis* and the sporadic distribution of the cluster among distantly related streptomycetes are consistent with the hypothesis that the kinanthraquinone B cluster was acquired by the *S. marxii* genome via horizontal gene transfer (HGT). Actinomycete integrative and conjugative elements are known to be capable of transferring up to a third of a chromosome between sympatric strains, thereby contributing to an evolutionary advantage in recipient strains [39]. Mobile genetic elements were also detected in the vicinity of the kinanthraquinone locus in *S. marxii*, which is consistent with an HGT origin [40]. Kinanthraquinone B probably provides an ecological advantage in soil communities by allowing the producer to suppress the growth of competitors through a mechanism that does not trigger immediate activation of resistance pathways associated with the SOS response [41].

### 3.7. Mechanistic Basis of Antimicrobial Activity and Biosynthetic Pathway

Kinanthraquinones stand out among anthraquinones due to their distinctive mechanism of action. While classical anthracyclines (e.g., doxorubicin) and fluoroquinolones stabilise DNA breaks, kinanthraquinones act as catalytic inhibitors [13]. This explains the absence of the TurboRFP signal in our experiments with pDualrep2: the enzyme is blocked before the double-strand break stage, so the DNA-damage signal does not reach the cellular regulatory network. From a clinical perspective, catalytic inhibitors of topoisomerases are considered promising agents with potentially lower genotoxicity and cardiotoxicity compared to topoisomerase poisons. However, the main barrier to their use against Gram-negative pathogens is the low permeability of the outer membrane [42]. Our work with the *lptD* mutant clearly demonstrates that, when this barrier is overcome (for example, through chemical modification of the molecule or the use of delivery systems), kinanthraquinone B exhibits a potent antibacterial effect [43].

While the primary focus of this study is the polyphasic taxonomic characterisation and description of *S. marxii* as a novel species, identification of its specialised metabolites provides essential phenotypic context. Our data suggest that kinanthraquinone B is the main contributor to the observed antimicrobial activity. This conclusion is based on three lines of evidence: (1) the presence of a syntenic biosynthetic gene cluster (BGC) in the genome, (2) the detection of the corresponding molecular ion (*m*/*z* 275.0550, [M+H]^+^) in the active HPLC fractions, and (3) a unique SOS-silent inhibition profile in the pDualrep2 system, which serves as a diagnostic fingerprint for this chemical class. For the purposes of species description, this level of dereplication is sufficient to link the genomic potential of the strain to its expressed phenotype.

In addition to the kinanthraquinone B cluster, the *S. marxii* genome contains a number of other promising BGCs. This makes *S. marxii* a valuable target for metabolomic studies and cryptic gene-cluster activation strategies such as OSMAC (One Strain–Many Compounds) or heterologous expression [44]. The diversity of PKS systems (types I, II and III) found in a single strain indicates high metabolic plasticity and the ability of *S. marxii* to synthesise a wide range of natural products [45]. The identification of *S. marxii* as a new species broadens our understanding of the biogeography of the genus *Streptomyces* and highlights the importance of preserving the microbial biodiversity of Russian soils as a source of innovative therapeutic agents.

### 3.8. Future Research Perspectives

The specific activity of *S. marxii* metabolites against the *E. coli lptD* mutant indicates the antibacterial potential of kinanthraquinone B, provided that the outer-membrane barrier can be bypassed. Future investigations will focus on expanding the screening of these metabolites against a broader range of biological targets. Due to the SOS-silent mechanism of catalytic topoisomerase inhibition, the activity of *S. marxii* extracts against Gram-positive pathogens and mycobacteria, which lack the restrictive outer membrane, is of particular interest. Furthermore, as some members of the kinanthraquinone family have demonstrated cytotoxic and antimalarial properties, future work will include testing against various tumour cell lines to fully assess the pharmacological potential and selectivity of this novel species’ metabolome.

### 3.9. Significance of S. marxii as a Novel Species: Comparative Perspective

The polyphasic characterisation of *S. marxii* sp. nov. places it within a distinct phylogenetic lineage that, despite sharing high 16S rRNA gene identity (>98%) with its closest relative *S. maoxianensis*, is clearly separated at the whole-genome level (ANI 92.53%, dDDH 47.9%), both values well below the accepted species demarcation thresholds of 95–96% and 70%, respectively. This discordance between 16S rRNA similarity and genome-level divergence exemplifies a known limitation of single-locus phylogeny in the genus *Streptomyces* [38], where intense recombination and horizontal gene transfer can decouple ribosomal and metabolic evolution. At the phenotypic level, *S. marxii* is distinguished from *S. maoxianensis* by its broader carbon source utilisation profile: D-mannitol, sucrose, L-arabinose and L-rhamnose are all assimilated by *S. marxii* but not by *S. maoxianensis* (Table 2). These differences in metabolic flexibility likely reflect adaptation to the organic-rich loam soils of the Yaroslavl Region and further support the species-level distinction.

Most significantly, the genome of *S. marxii* encodes a complete T2PKS biosynthetic cluster for kinanthraquinone B that is entirely absent in *S. maoxianensis* and sporadically distributed among distantly related streptomycetes. This genomic divergence translates into a unique functional phenotype unobserved in related taxa: an SOS-silent antimicrobial activity consistent with catalytic topoisomerase inhibition. From a biotechnological perspective, *S. marxii* represents a natural producer of a rare chemical scaffold with a mechanism that may circumvent conventional resistance-induction pathways associated with the SOS response. The strain’s broader BGC repertoire—comprising PKS systems of types I, II and III—further positions it as a high-value candidate for metabolomic profiling and cryptic cluster activation strategies. Together, these findings demonstrate that the formal description of *S. marxii* sp. nov. contributes a biologically and pharmacologically distinct entity to the known diversity of the genus *Streptomyces*.

## 4. Conclusions

In this study, *S. marxii*, isolated from soil samples in the Yaroslavl Region, was comprehensively characterised using modern genomic and mass-spectrometric methods. Based on ANI (92.53%), dDDH (47.9%) and taxonomic profiling, the strain was classified as a representative of a new species of the genus *Streptomyces*, phylogenetically closest to *S. maoxianensis*. Kinanthraquinone B ([M+H]^+^ *m*/*z* 275.0550) was identified as the main biologically active metabolite of the strain. Functional screening on *E. coli* reporter strains confirmed a catalytic inhibition of DNA topoisomerases II and IV that does not induce the SOS response in bacterial cells. Genomic analysis indicates that the kinanthraquinone biosynthetic cluster was acquired by the strain via horizontal gene transfer, highlighting the role of chromosomal plasticity in the adaptation of soil microorganisms. *S. marxii* is a promising producer of rare antibiotics with an SOS-silent mechanism of action, and its genome contains a significant reserve for the discovery of new specialised metabolites.

## 5. Description of *Streptomyces marxii*

*Streptomyces marxii* (marx.i’i. N.L. gen. masc. n. *marxii*, named in honour of Karl Marx).

Gram-positive, aerobic, non-motile actinobacterium. Forms branched substrate mycelium (yellowish-brown) and abundant aerial mycelium (greyish-white to light grey on ISP 3). Spore chains are long and flexible, with 10–50 ellipsoidal spores. Mesophilic: growth at 15–37 °C (optimum 28 °C). Neutrophilic: pH 5.5–8.5 (optimum 7.0). Halotolerant: up to 5% (*w*/*v*) NaCl. Utilises D-glucose, D-mannitol, sucrose, L-arabinose, D-galactose, D-xylose, D-mannose, L-rhamnose, D-raffinose, D-fructose and *myo*-inositol; does not utilise lactose, cellobiose or salicin. Produces pigments on ISP 6 and ISP 7. Cell wall predicted to contain LL-diaminopimelic acid and glycine. Predicted major menaquinones: MK-9(H_6_), MK-9(H_8_). Predicted major branched-chain fatty acids based on genomic annotation include iso-C_16:0_ and anteiso-C_15:0_.

The type strain, VKM Ac-3100^T^, was isolated from soil in the Yaroslavl Region, Russia. The genome has a G+C content of 69.6 mol%. The GenBank accession number for the 16S rRNA gene is PV849038; the whole-genome sequence has been deposited in GenBank under accession number SAMN57460202.

## Figures and Tables

**Table 1:** Cultural characteristics of *S. marxii* (14 days of incubation).

Medium	Growth	Aerial Mycelium (RAL)	Substrate Mycelium (RAL)	Soluble Pigment
ISP 2	Abundant	1014 Ivory	1014 Ivory	None
ISP 3	Abundant	9002 Grey white	8008 Olive brown	8001 Ochre brown
ISP 4	Moderate	1001 Beige	1014 Ivory	None
ISP 5	Moderate	8008 Olive brown	8008 Olive brown	1036 Pearl gold
ISP 6	Well	8007 Fawn brown	8007 Fawn brown	None
ISP 7	Well	8001 Ochre brown	8001 Ochre brown	8001 Ochre brown

**Table 2:** Carbon source utilisation profiles of *S. marxii* and *S. maoxianensis. *+, growth on the carbon source (utilised); -, no growth (not utilised).

Carbon Source	*S. marxii*	*S. maoxianensis* NEAU-Spg16
D-Glucose	Used (+)	Used (+)
D-Mannitol	Used (+)	Not used (-)
Sucrose	Used (+)	Not used (-)
L-Arabinose	Used (+)	Not used (-)
D-Galactose	Used (+)	Used (+)
Myo-inositol	Used (+)	Used (+)
D-Xylose	Used (+)	Used (+)
D-Mannose	Used (+)	Used (+)
L-Rhamnose	Used (+)	Not used (-)
D-Raffinose	Used (+)	Used (+)
D-Fructose	Used (+)	Used (+)
Lactose	Not used (-)	Not used (-)
Cellobiose	Not used (-)	Not used (-)
Salicin	Not used (-)	Not used (-)

**Table 3:** Genomic indices (dDDH and ANI) and general characteristics of the *S. marxii* genome compared with those of the most closely related type strains. dDDH and ANI values were calculated using the TYGS platform [17], which is based on the Genome-to-Genome Distance Calculator (GGDC 3.0) [33].

Strain	dDDH (%)	ANI (%)	Size (Mb)	G+C Content (%)	Accession No.
*S. marxii*	100.0	100.0	8.99	69.6	PV849038
*S. maoxianensis* CGMCC 4.7139	47.9	92.53	7.81	69.6	GCA_042654885
*S. lunaelactis* DSM 42149	34.4	88.83	8.57	69.8	GCF_003054555
*S. achmelvichensis* MS2.AVA.5	28.2	84.15	9.63	70.0	GCF_037892235
*S. xantholiticus* JCM 4863	26.1	82.52	7.75	70.5	GCA_014651015
*S. kurssanovii* JCM 4388	26.0	82.31	8.16	70.9	GCA_014649695
*S. formicae* DSM 100524	25.5	81.94	8.21	70.8	GCA_019614655
*S. chengmaiensis* HNM0663	25.4	81.82	7.96	71.0	GCF_029846775
*S. pratisoli* MS1.AVA.4	25.2	81.71	8.61	70.8	GCF_037860615
*S. vilmorinianum* YP1	24.8	81.44	7.65	71.6	GCA_005517195
*S. lateritius* JCM 4389	24.4	81.18	7.69	71.3	GCA_014649715
*S. gelaticus* JCM 4376	24.3	81.02	7.73	70.6	GCA_014649535
*S. helvaticus* JCM 4768	24.3	81.01	7.65	71.4	GCA_014650855
*S. chryseus* JCM 4737	24.1	80.95	7.12	71.6	GCA_014650755
*Kitasatospora cinereorecta* JCM 6916	24.0	80.84	7.23	71.0	GCA_042652405
*S. badius* JCM 4350	23.7	80.62	7.45	71.7	GCA_014649415
*S. rubiginosohelvolus* JCM 4415	23.6	80.55	7.54	71.7	GCA_014649875
*S. nashvillensis* JCM 4498	23.7	80.61	8.58	72.3	GCA_014650095
*S. gulbargensis* JCM 16956	23.6	80.54	6.76	73.1	GCA_039537365

## Data Availability

The original data presented in the study are openly available in GenBank. The GenBank accession number for the 16S rRNA gene sequence of *S. marxii* VKM Ac-3100^T^ is PV849038; the whole-genome sequence has been deposited under accession number SAMN57460202.

## Abbreviations

The following abbreviations are used in this manuscript: ANI, average nucleotide identity; BGC, biosynthetic gene cluster; dDDH, digital DNA–DNA hybridisation; GBDP, Genome BLAST Distance Phylogeny; GGDC, Genome-to-Genome Distance Calculator; HGT, horizontal gene transfer; HPLC, high-performance liquid chromatography; HRMS, high-resolution mass spectrometry; ISP, International Streptomyces Project; LL-DAP, LL-diaminopimelic acid; MDR, multidrug-resistant; MIBiG, Minimum Information about a Biosynthetic Gene Cluster; OSMAC, One Strain–Many Compounds; SEM, scanning electron microscopy; SOS response, SOS DNA-damage response; SPE, solid-phase extraction; T2PKS, type II polyketide synthase; TYGS, Type Strain Genome Server; WGS, whole-genome sequencing.
